# Patients with Lung Cancer of Different Racial Backgrounds Harbor Distinct Immune Cell Profiles

**DOI:** 10.1158/2767-9764.CRC-22-0057

**Published:** 2022-08-29

**Authors:** Yitian Xu, Licheng Zhang, Jose Thaiparambil, Sunny Mai, Dimuthu Nuwan Perera, Jilu Zhang, Ping-Ying Pan, Cristian Coarfa, Kenneth Ramos, Shu-Hsia Chen, Randa El-Zein

**Affiliations:** 1 Houston Methodist Research Institute, Houston, Texas.; 2 Immune Monitoring core, Houston Methodist Research Institute, Houston, Texas.; 3 Dan L Duncan Comprehensive Cancer Center, Center for Precision Environmental Health, Department of Molecular and Cellular Biology, Baylor College of Medicine, Houston, Texas.; 4 Institute of Biosciences and Technology, Texas A&M University, Houston, Texas.

## Abstract

Tumors accumulated with infiltrated immune cells (hot tumors) have a higher response rate to immune checkpoint blockade, when compared with those with minimal T-cell infiltration (cold tumors). We report here that patients with lung cancer with different racial backgrounds harbored distinct immune cell profiles in the tumor microenvironment. Compared with African Americans (AA), Caucasian Americans (CA) exhibited increased immune cell infiltration and vasculature, and increased survival. Changes of survival and immune profile were most pronounced among active smokers and nonsmokers, compared with former smokers and total patients. Neighborhood analysis showed that immune cells accumulated around cancer cells in CAs but not AAs. Our findings reveal intrinsic biological differences between AA and CA patients with lung cancer, suggesting that treatment plans should be tailored for patients with different racial backgrounds.

**Significance:**

We report biological racial differences among patients with lung cancer where Caucasians present a hot tumor microenvironment compared with cold tumor in AAs. Treatment plans should be customized to maximize therapeutic outcomes.

## Introduction

Lung cancer, the leading cause of cancer mortality in the United States accounts for more deaths each year than colon, breast, prostate, and pancreatic cancers combined (1, 2). In 2019, 25,000 new lung cancer cases were diagnosed in African Americans (AA) with 6,550 deaths, the highest lung cancer death rate of any racial or ethnic group (2–4). Survival is lower in AA patients at every stage of diagnosis, with a 5‐year relative survival rate of 16% in AAs versus 19% in Caucasian Americans (CA; ref. 2). Socioeconomic barriers are recognized as the primary contributors; however, studies report that racial disparities persist even after accounting for socioeconomic factors and barriers to care (5, 6). A recent study showed higher death rates in AAs versus CAs within the same socioeconomic strata (7), suggesting that intrinsic biological factors may also contribute to these disparities. Furthermore, AAs develop chronic obstructive pulmonary disease **(**COPD) at a younger age despite less cumulative smoking, suggesting greater susceptibility (8). Evasion of the host immune response may also play an important role in lung cancer and COPD (9), with differential gene expression and M1-like and M2-like macrophage infiltration profiles in lung cancer in AAs versus CAs (10).

The immune system plays an important role in the development and progression of cancer (11). T-cell activation eliminates cancer cells and promotes tumor regression (12, 13). Increased tumor-inflitrating T cell is a hallmark of active immune response against tumor and has been associated with improved prognosis (12, 13). On the other hand, cancer cells can induce an immune-suppressive microenvironment (14, 15) by recruiting suppressive cells such as regulatory T cells (Treg) and myeloid-derived suppressor cell (MDSC), which cause T-cell exhaustion and/or senescence. Smoking-induced tissue inflammation correlates with macrophage and MDSC infiltration into the lung (16). Tumors with ample T-cell inflitration and proinflammatory cytokine accumulation are commonly termed “hot tumors” (12, 13, 17). Hot tumors, such as melanoma or some lung cancers, demonstrate an increased response to immune checkpoint inihibitors. In contrast, “cold tumors” are characterized by low immune cell infiltration (with mostly immune-suppressive cells) or non–T-cell infiltration, as seen with prostate and pancreatic cancers (12, 13, 17). However, it remains to be determined how these immune cell populations differentially regulate lung cancer development in patients across different racial backgrounds, disease stages or inflammatory status.

In this study, we identified intrinsic differences between AA and CA patients with lung cancer. CA patients had better survival compared with AA patients, which may result from increased tissue inflammation and elevated immune populations infiltrated into the tumor in CA patients. More specifically, CA patients had significantly greater numbers of CD4 and CD8 T cells, as well as increased vasculature within the tumor, indicating a more active immune response against tumor progression. Interestingly, we found a striking difference in survival between AA and CA patients, for both active cigarette users and nonsmokers. This observation was consistent with their immune profiles where greater numbers of CD4 and CD8 T cells were detected among active smokers or nonsmokers CAs compared with AAs. Moreover, neighborhood analysis of the tumor microenvironment revealed that cancer cells in CA patients were surrounded by immune cells; while in AA patients, these immune cell populations avoided the cancer cells. These data demonstrate a “hot tumor” microenvironment in CA patients with lung cancer and a “cold tumor” microenvironment in AA patients with lung cancer, which may define different clinical trajectories for these patient populations.

## Methods and Materials

### Patient Cohort

A total of 157 patients with lung cancer were enrolled in Houston Methodist hospital since 2015. All patients provided written informed consent for using their tissues for Institutional Review Board (IRB)-approved research. The study was conducted in accordance with the Declaration of Helsinki and the International Conference on Harmonization Guidelines for Good Clinical Practice. Records of patient information were obtained under approval of IRBs. Among these patients, tissue from 55 patients with paired tumor and adjacent normal tissue and 21 patients with only tumor tissue was stored at the Houston Methodist Biorepository. Patient demographics are shown in Table 1.

**TABLE 1:** Patient demographics.

Race	
CA	94/157 (59.87%)
AA	63/157 (40.13%)
Sex	
Male	70/157 (44.59%)
Female	87/157 (55.41%)
Tumor type	
Adenocarcinoma	108/155 (69.68%)
Squamous carcinoma	47/155 (30.32%)
Smoking history	
Current smoker	35/145 (24.14%)
Former smoker	84/145 (57.93%)
Nonsmoker	26/145 (17.93%)
Age (at diagnosis)	
45–54	10
55–64	36
65–74	73
>75	42
Disease stage	
IA	72
IB	28
I (unspecified)	6
IIA	14
IIB	7
IIIA	9
IV	2

### RNA Sequencing

Lung adenocarcinomas and lung squamous cell carcinomas and paired adjacent normal frozen tissue samples (15 each subtype) were obtained from the Houston Methodist biorepository under IRB-approved protocol. These included 12 female and 18 male samples with an age range of 54–80 years. Tumor versus normal adjacent tissue designations in hematoxylin and eosin slides were made by a trained pathologist. RNA extracted from fresh frozen tissue by laser capture microdissection was sent to the Sequencing and Gene Editing Core at the University of Houston for: RNA quality analysis using the Agilent 2100 Bioanalyzer; sequencing library preparation using the Illumina Truseq stranded total RNA kit, with 1,000 ng of RNA per sample with a RNA integrity number (RIN) value >8.4; and sequencing using the NextSeq 500 (Illumina). RNA sequencing (RNA-seq) data were mapped onto the human genome UCSC hg19 using hisat2 (18), and gene expression quantified using StringTie and GENCODE model. Differentially expressed genes were detected using unequal variance two-sided Student *t* test with the Benjamini–Hochberg correction, with significance achieved at FDR < 0.05 and fold change exceeding 1.25 ×. Enriched pathways were inferred using the hypergeometric test, with significance achieved for FDR < 0.05.

### Imaging Mass Cytometry

Methods of conjugating metal-labeled antibodies, tissue staining, and data analysis were described previously (19, 20). Each antibody was individually validated using appropriate positive controls and titrated for a suitable concentration without signal overspill. Briefly, antibodies in buffer without BSA or carrier protein were conjugated to metals of interest using the MaxPar antibody conjugation kit (Fluidigm). Antibodies used in our study are listed in Supplementary Table S1. Of 55 patients with paired tumor and adjacent normal tissue and 21 patients with only tumor tissue, 26 patients were selected on the basis of different tumor subtypes (Supplementary Table S2) and disease stages (Supplementary Table S3) for tissue sectioning. For tissue staining, tumor sections were baked at 60°C overnight, then dewaxed in xylene and rehydrated in a graded series of alcohol. Heat-induced epitope retrieval was performed in a EZ-Retriever System (BioGenex) at 95°C in Tris-Tween20 buffer at pH 9 for 20 minutes. After immediate cooling for 20 minutes, the sections were blocked with 3% BSA in TBS for 1 hour, and then incubated overnight at 4°C with an antibody master mix (Supplementary Table S1). Samples were then washed four times with TBS/0.1% Tween20 before staining with Cell-ID Intercalator (Fluidigm) for 5 minutes for nuclear staining. Slides were washed twice with TBS/0.1% Tween20 and air dried to store at 4°C for ablation.

The sections were ablated with Hyperion system (Fluidigm) for data acquisition. Imaging mass cytometry data were segmented by ilastik and CellProfiler. Histology topography cytometry analysis toolbox (HistoCAT) and R scripts were used to quantify cell number, generate tSNE plots, and perform neighborhood analysis. Cell density of each population was plotted in Prism. Neighborhood analysis was compared between AA and CA patients. Each block represents cluster X in *X* axis is surrounded (colored in red) or avoided (colored in blue) by cluster Y in *Y* axis. Change of cluster relationship was identified between AA and CA patients.

### Statistical Analysis

Statistical analysis was performed in Prism (Graphpad) except the correlation analysis. *P* values were calculated by unpaired *t* test unless stated otherwise in figure legends. For correlation analysis, the trend of increase or decrease from CA and AA patients was calculated, and the *P* values were calculated in R scripts to determine significance of difference between these trends.

### Data Availability Statement

The RNA-seq data underlying this article are available in the NCBI Gene Expression Omnibus at http://www.ncbi.nlm.nih.gov/geo/ and can be accessed with accession number GSE159857.

## Results

### Differential Gene Expression Between AA and CA Patients with Non–small Cell Lung Cancer

To elucidate differences in tumor biology between AAs and CAs, we performed RNA-seq analysis on tumor and adjacent normal tissues from 30 patients with non–small cell lung cancer (NSCLC) (15 adenocarcinoma and 15 squamous cell carcinoma). After removing samples with low-quality RNA-seq data, the final analysis included 11 tumor tissues for adenocarcinoma and 12 tumor tissues for squamous cell carcinoma. In terms of race distribution, the patients included 5 AAs (2 adenocarcinoma and 3 squamous cell carcinoma) and 18 CAs (9 adenocarcinoma and 9 squamous cell carcinoma).

**FIGURE 1:**
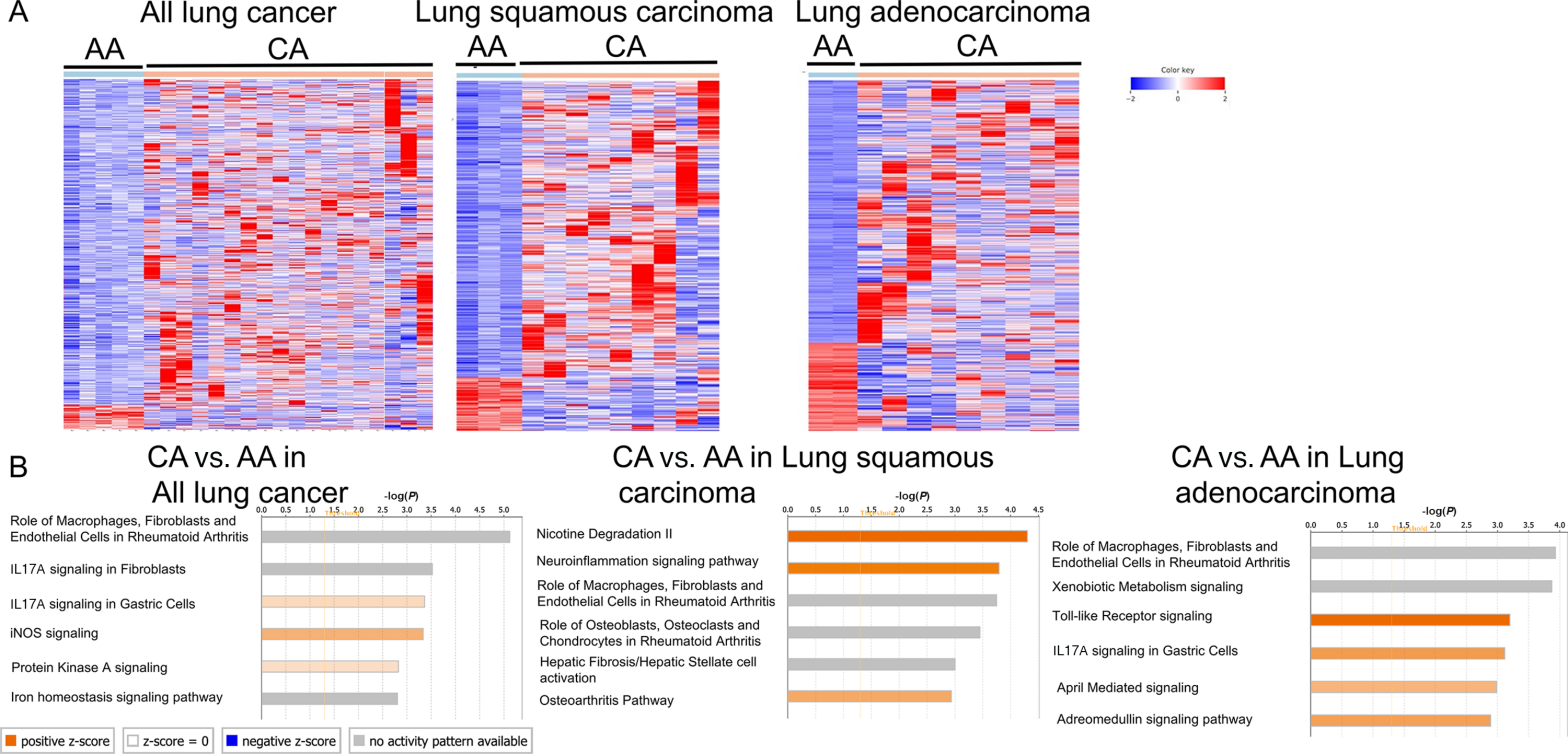
Distinct gene expression patterns between AA and CA patients with NSCLC. **A,** Transcriptome analysis of lung tumor versus adjacent normal tissue from 5 AA (cyan bar) and 18 CA (orange bar) patients. **B,** Pathway analysis by normalizing CA versus AA in all lung cancer types (left), in lung squamous carcinomas (middle), or lung adenocarcinomas (right).

Significant differences in gene expression profiles between AAs and CAs were identified (Fig. 1A), with 777 genes upregulated and 56 genes downregulated (*P* < 0.05). Among the significantly different genes identified, *IL1A*, *IL1B*, *MYD88*, *TLR4*, *NFKB1* were highly upregulated in CA patients compared with AA patients, suggesting increased immune cell activation in CAs. In addition, elevated levels of *CXCL11*, *CCL2*, *CX3CR1* indicated increased recruitment of immune cells. Pathway analysis confirmed increased immune activation in CAs compared with AAs (Fig. 1B), with patients with adenocarcinoma showing increased immune activation pathways including Toll-like receptor pathways and IL17A signaling in CAs compared with AAs. Increased activity of nicotine degradation pathways was seen in CA patients with squamous cell carcinoma compared with AA patients, suggesting an elevated response against the tissue damage caused by nicotine. These data indicate distinct tumor immune landscapes between AA and CA patients with lung cancer.

### Increased Patient Survival and Immune Cells in CA Patients with NSCLC

**FIGURE 2:**
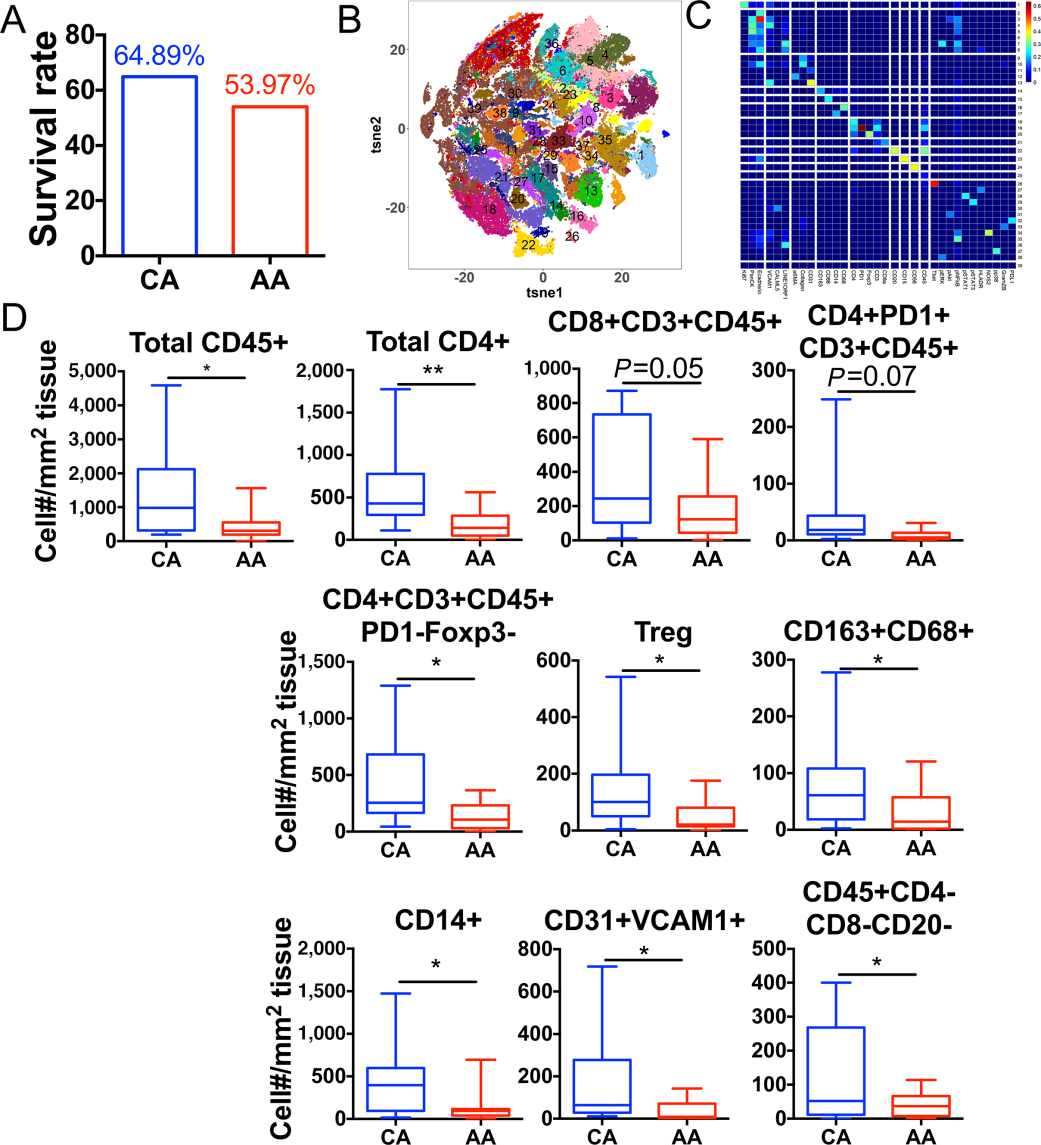
Immune microenvironment in NSCLC is distinct between patients of different racial backgrounds. **A,** Lung cancer patient survival rate from a cohort of 157 patients segregated by race. Imaging mass cytometry was performed on 26 samples (12 AA and 14 CA patients) to generate tSNE plot (**B**), phenograph heatmap (**C**), and cell density of different populations (**D**). **B,** Number on tSNE indicates the clusters identified. **C,***X* axis shows the 35 markers used in Supplementary Table S1. *Y* axis shows the 38 populations (Table 2) identified. Unpaired Student *t* test. *, *P* < 0.05. **, *P* < 0.01.

To further validate our RNA-seq findings, we obtained information and tissues from a cohort of 157 patients with lung cancer from the Houston Methodist Biorepository. Tissues were obtained from 24 AA and 52 CA patients with lung cancer treated at Houston Methodist Hospitals. AA patients showed poorer overall survival when compared with CA patients (53.97% vs. 64.89%, respectively; Fig. 2A). To determine whether the increased survival in CA patients is associated with increased immune infiltration, as suggested by RNA-seq, we sampled a subgroup of 26 patients (12 AA patients and 14 CA patients at different stages of lung cancer) and performed imaging mass cytometry (IMC) to assess their immune profiles (Fig. 2B and C). The unsupervised clustering algorithm generated a phenograph heatmap based on marker profiles (Fig. 2C) and identified 38 cell clusters as shown in the tSNE map (Fig. 2B; Table 2).

**TABLE 2:** Clusters identified by IMC analysis.

Cluster	Celltype
1	Ki67^+^
2	Ecad^+^
3	Ecad^+^PanCK^+^VCAM1^+^pNFκB^+^pAkt^+^
4	PanCK^+^VCAM1^+^pNFκBlo
5	PanCK^+^
6	PanCK^+^Ecad^+^
7	pNFκB^+^LINE1^+^pERK^+^
8	Ecad^+^LINE1^+^
9	Collagen^+^
10	Collagen^+^Ecad^+^
11	CD31^+^
12	aSMA^+^
13	CD31^+^VCAM1^+^
14	CD163^+^CD68^+^
15	CD86^+^
16	CD68^+^
17	CD14^+^
18	CD4^+^CD3^+^CD45^+^
19	CD4^+^PD1^+^CD3^+^CD45^+^
20	CD4^+^Foxp3^+^
21	CD8^+^CD3^+^CD45^+^
22	CD20^+^CD45^+^
23	CD15^+^
24	CD56^+^
25	CD45^+^
26	Tbet^+^
27	HLADR^+^
28	pSTAT1^+^
29	pSTAT3^+^
30	CALML5^+^
31	pAkt^+^
32	PDL1^+^
33	GramZB^+^
34	NOS2^+^
35	pNFκB^+^
36	LINE1^+^
37	pp38^+^
38	pERK^+^
39	Surface^−^

An elevated level of total CD45^+^ cells was identified in CAs (Fig. 2D), denoting increased numbers of immune cells in the tumor microenvironment. This observation is found within patient tumor tissue, but not in the normal adjacent tissue, which has relatively few immune cells (Supplementary Fig. S1). More specifically, CD4^+^ T cells and CD8^+^ T cells were significantly increased in CAs, as were CD14^+^ monocytes and other less common immune cells, that is, cells without CD4/CD8/CD20/CD14 expression (Fig. 2D). In addition, inactivated CD4^+^ T cells (CD4^+^CD3^+^CD45^+^PD-1^−^Foxp3^−^) and activated CD4^+^ T cells (CD4^+^CD3^+^CD45^+^PD-1^+^) were in higher abundance in CA patients, suggesting a stronger CD4 T-cell response and a higher potential of incoming CD4 T-cell response in CA patients. Interestingly, there were more immune-suppressive Treg and M2-like macrophages (CD163^+^CD68^+^) in CAs, possibly to counter balance the immune response associated with increased CD4 and CD8 T cells. Together, these observations indicate a “hot tumor” microenvironment in CA patients, compared with a “cold tumor” microenvironment in AA patients. In keeping with this interpretation, CA patients exhibited higher numbers of CD31^+^ endothelial cells, indicating increased vasculature in the “hot tumor” microenvironment. In summary, CA patients with lung cancer had increased immune cells and vasculature present within their tumor microenvironment. This pattern is consistent with the increased survival seen among CAs.

### Smoking Contributes to Differences in Survival and Immune Profile Between AA and CA Patients with NSCLC

**FIGURE 3:**
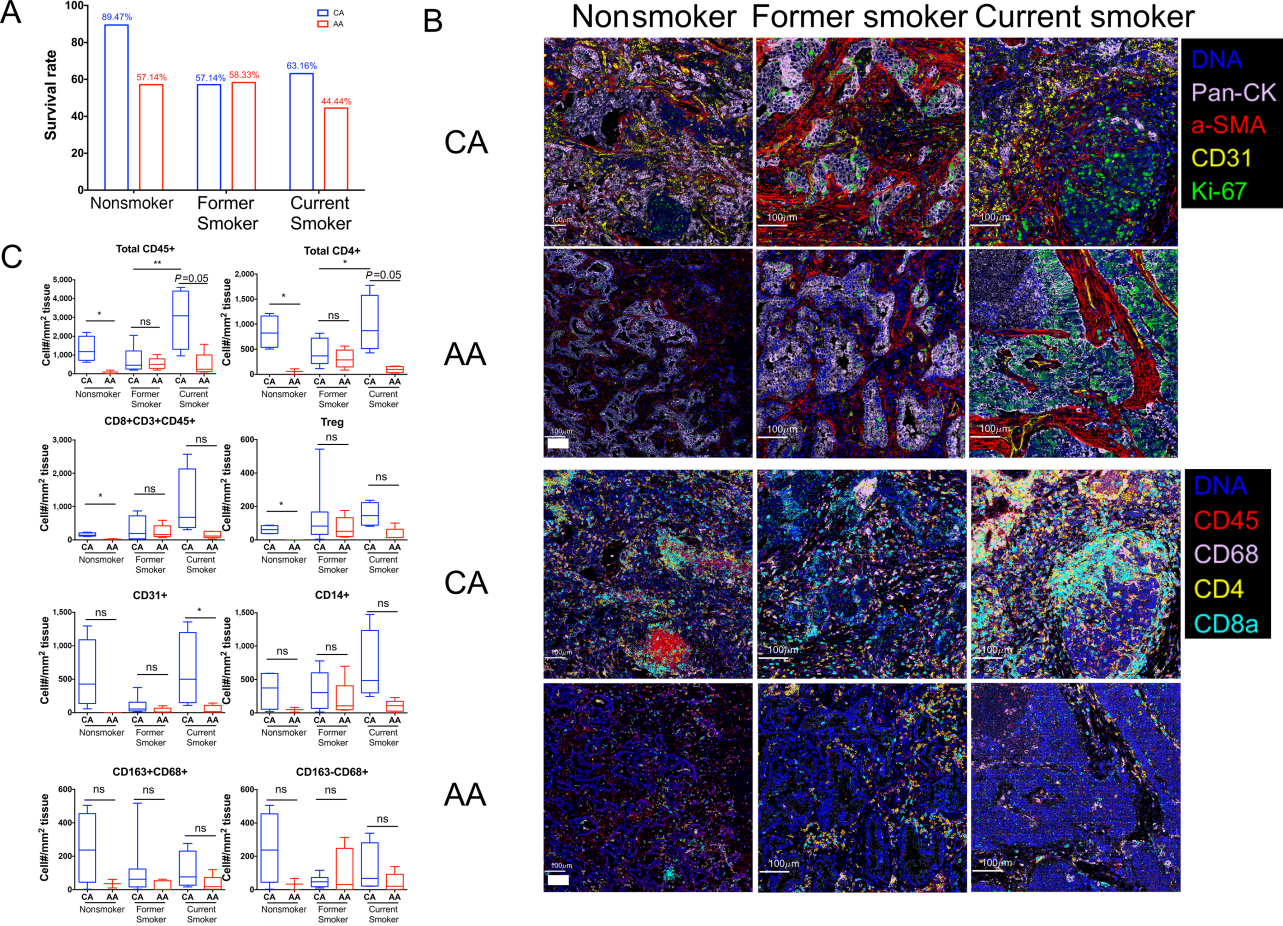
Different immune microenvironment in NSCLC is associated with patients’ smoking history. **A,** Lung cancer patient survival rate stratified by smoking history. **B,** Overlaid images of tissue stained by imaging mass cytometry from 12 AA and 14 CA patients of different racial backgrounds and smoking histories. Scale bar: 100 μm. **C,** Cell density of populations identified by IMC stratified by patients’ smoking history. CA versus AA: unpaired Student *t* test. *, *P* < 0.05. Nonsmoker versus former smoker versus current smoker: one-way ANOVA test.

Cigarette smoking is the major cause of lung cancer. Between 50% and 80% of patients with lung cancer have preexisting COPD, compared with a 15% to 20% prevalence of COPD in the general smoking population (4, 21, 22), and studies have shown that smokers with COPD are at increased risk for developing lung cancer (2, 23). We further stratified the survival data of the 157 patients with lung cancer based on their smoking history (Fig. 3A). Intriguingly, within the current smokers, AA patients have considerably lower survival rates compared CA patients (44.44% vs. 63.16%). Within the former smoker category, survival rates were comparable between AA patients and CA patients (58.33% vs. 57.14%). Within nonsmokers, CA patients had the highest survival rate, compared with AA patients (89.47% vs. 57.14%). These findings are consistent with other studies showing that cigarette smoking can induce lung cancer and impair patients’ survival (1, 24, 25). Our results further reveal survival differences between AA and CA patients with different smoking histories.

Among both current smokers and nonsmokers, we also observed significantly higher numbers of CD45^+^ cells in CA patients (Fig. 3B and C). Similarly, total CD4^+^ T cells, CD8^+^ T cell, and CD31^+^ endothelial cells were increased in CA patients who were current smokers and nonsmokers, but not in former smokers (Fig. 3B). Although not statistically significant, CA patients showed higher abundance of CD14^+^ monocytes and M2-like macrophages (CD163^+^CD68^+^) among current smokers. However, this increase was not observed in former smokers, that is, CA patients had similar numbers of immune cells compared with the AA patients, consistent with similar survival rates. These findings are consistent with survival outcomes in these patient subpopulations (Supplementary Fig. S2).

Among patients of same race, comparisons between nonsmokers, former smokers and current smokers were also performed. Significant difference was only found in CD45^+^ cells and CD4^+^ cells between former smokers and current smokers of CA patients (Fig. 3C). We also investigated other factors that might contribute to differences of survival and immune profile between CA and AA patients, such as sex, tumor type, survival status, and disease stage. However, these factors had minimal to no effect on the immune cell profile (Supplementary Figs. S2–S7). To sum up, CA patients with lung cancer who were active smokers or nonsmokers had better survival and increased intratumor immune cell infiltration compared with AA patients.

### Spatial Relationship Between Immune Cells and Cancer Cells Reveals Distinct Tumor Microenvironments Between AA and CA Patients with NSCLC

**FIGURE 4:**
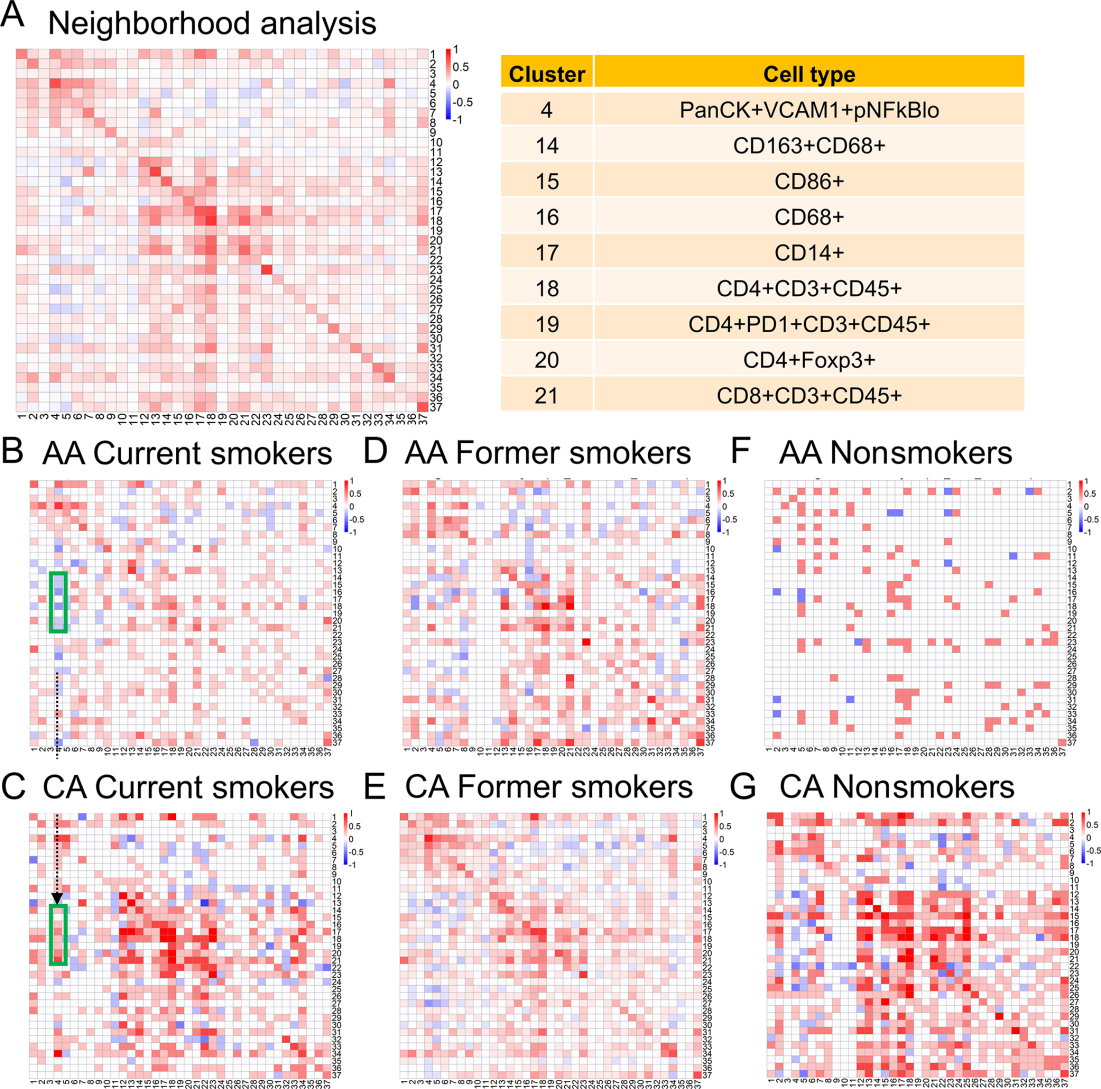
Neighborhood analysis reveals the spatial relationship between different cell populations within patient tumor tissue. Each block represents clusters that on the *X* axis are surrounded (colored in red) or avoided (colored in blue) by clusters on the *Y* axis. All patients were clustered for analysis (**A**) and then separated for CA (**C**, **E**, **G**) and AA patients (**B**, **D**, **F**) based on their smoking histories. **B** and **C**: current smokers. **D** and **E**: former smokers. **F** and **G**: nonsmokers. Selected cell clusters are presented in **A**. Highlighted clusters within green boxes indicate the different spatial relationship between selected immune cell clusters (**A**) and cancer cells in AA (**B**, avoidance) and CA patients (**C**, surrounding).

We next performed neighborhood analysis of the tumor microenvironment to assess the spatial relationship between the cell populations identified (Fig. 4). Each block represents clusters in the *X* axis that are surrounded (colored in red) or avoided (colored in blue) by clusters in the *Y* axis. We divided the data into different groups based on smoking history and compared AA and CA patients. Considering that the white blocks (neither surrounding nor avoidance) could result from low sample numbers in some groups, we looked for changes in immune cell populations between AA and CA patients with opposite relationships. Interestingly, we found that cancer cells (cluster 4, PanCK^+^E-cadherin^+^VCAM1^+^ pNFκB^+^) from current smoker AA patients (Fig. 4B) were avoided by endothelial cells (cluster 14, CD31^+^VCAM1^+^) and several immune populations (Fig. 4B, green box), including macrophages (cluster 16), CD4 T cells (cluster 18), Tregs (cluster 20), and CD8 T cells (cluster 21). This suggests a “cold tumor” microenvironment, where immune cells are not in the vicinity of cancer cells. On the other hand, cancer cells (cluster 4, PanCK^+^E-cadherin^+^VCAM1^+^ pNFκB^+^) from current smoker CA patients (Fig. 4C) were surrounded by endothelial cells (cluster 14, CD31^+^VCAM1^+^) and these immune populations (Fig. 4C, green box), including macrophages (cluster 16), CD4 T cells (cluster 18), Tregs (cluster 20), and CD8 T cells (cluster 21). This establishes a “hot tumor” environment within the CA patients and further explains survival differences (Fig. 3A). We did not observe a different relationship between these populations within former smokers (Fig. 3C), a finding consistent with their similarity in survival (Fig. 3A). However, we also did not observe a different relationship between these populations within nonsmokers (Fig. 3C), despite survival differences between AA and CA patients (Fig. 3A), which may be the result of limited sample numbers from nonsmoker AA patients. Nevertheless, our results demonstrate that in sharp contrast to CA patients, immune cells and endothelial cells are not surrounding the tumor cells in AA patients, suggesting a “cold tumor” microenvironment.

### Minimal Differences in Tumor Microenvironment Between AA and CA Patients with NSCLC at Early Disease Stages, that Become Significant as Disease Progresses

**FIGURE 5:**
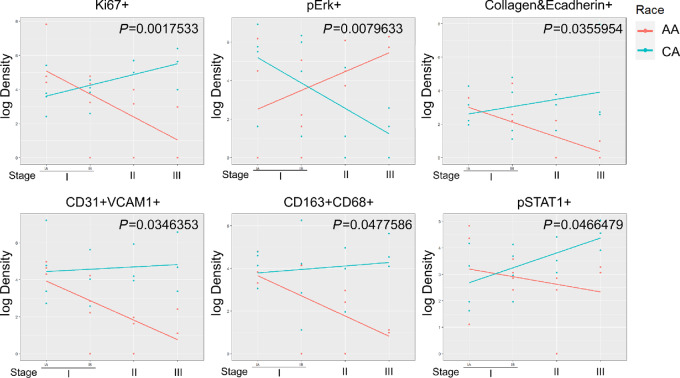
Correlation between cell clusters and disease stages in AA and CA patients are different. The cell density of each cluster at different disease stages was plotted in a log scale. Linear regression was used to fit each cluster (CA: cyan line, AA: red line). Clusters with significant *P* values (*P* < 0.05) between CA and AA patients were presented.

Next, we asked whether the cell clusters identified correlated with disease stages in a different pattern between AA and CA patients. We divided these 26 patients based on disease stages and plotted the cell density at different disease stages on a log scale. Linear regression fitting each cluster found distinct patterns of correlation between AA and CA patients (Fig. 5). Ki67^+^ cells, collagen^+^E-cadherin^+^ cells, CD31^+^ endothelial cells, CD163^+^ macrophage, and pSTAT1^+^ cells were significantly increased in CA patients with advanced disease stages. While Ki67 stains for proliferative cells, with the majority of Ki67^+^ cells expected to be tumor cells, this relationship only held true for CAs and not AAs (Fig. 5). This difference may be partly accounted for by differences between the hot versus cold tumor microenvironments in CA versus AA patients. In the hot tumor microenvironment, tumor cells may need to compensate for increased killing by the immune cells. In addition, the Ki67 readout was derived from subjects at different stages and with varying confounding by sex, smoking history, tumor types, and treatment.

AA patients showed a significant decrease in these populations with increased disease severity. Conversely, CA patients displayed a significantly decreased correlation between pERK^+^ cells and disease severity, while AA patients had an increased correlation between these cells. Consistent with earlier findings establishing a “hot tumor” environment in CA patients and a “cold tumor” environment in AA patients, we found increasing differences of endothelial cells and M2-like macrophages as the disease progressed to late stages (Fig. 5). More interestingly, most of these clusters (5/6) showed similar numbers between AA and CA patients at the earliest stage of disease, with differences becoming more significant with disease progression (Fig. 5). This indicates that AA and CA patients have minimal differences in their tumor environment at early stages. As the disease progresses, differences were amplified and potentially impact patient survival. In summary, AA and CA patients with lung cancer display distinct correlation patterns between cell populations and disease severity, especially during late stages of disease, which could affect patient survival and treatment outcomes.

### Long Interspersed Element-1 Expression Among Patients with NSCLC

**FIGURE 6:**
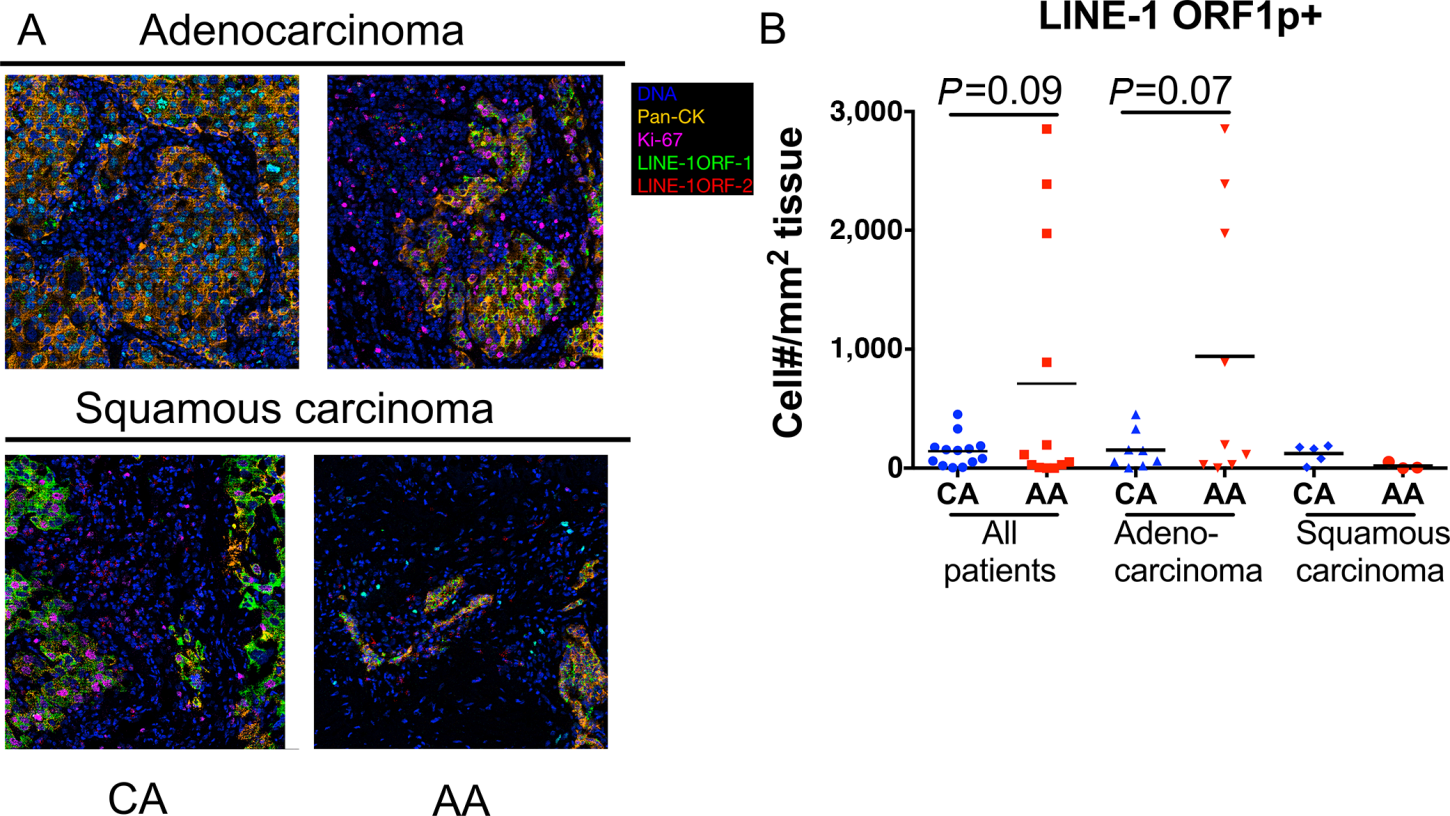
LINE-1 expression in patients with NSCLC. **A,** LINE-1 expression in CA and AA patients with adenocarcinoma or squamous carcinoma. **B,** LINE-1 positive cells in CA and AA patient tumor tissue. Unpaired Student *t* test. *, *P* < 0.05.

During tumorigenesis, immune cells can be recruited and altered by cancer cells to create an immune-suppressive environment. This process is influenced by long interspersed element-1 (LINE-1) for its ability to regulate cell metabolism, tissue inflammation, and extracellular matrix deposition (26–31). Moreover, LINE-1 is associated with TGFβ1 levels (32), which skews the immune cells toward a suppressive phenotype (33–35). LINE-1 is the only retrotransposon that remains active in the human genome (36). Retrotransposition is a highly mutagenic process associated with genomic instability, aberrant regulation of DNA repair, altered extracellular matrix deposition, inflammation, and changes in cellular metabolism (37, 38). As we identified differences in immune profiles between CA and AA patients, we examined the expression level of LINE-1 ORF1p by IMC (Fig. 6A). We found more LINE-1 ORF1p^+^ cells in patients with adenocarcinoma compared with patients with squamous carcinoma (Fig. 6B). Within patients with adenocarcinoma, more LINE-1 ORF1p^+^ cells were observed in AA patients compared with CA patients (Fig. 6B), which may potentially contribute the lower survival rate (Fig. 2A). Interestingly, by dividing these patients based on tumor types, we found significantly more LINE-1 ORF1p^+^ cells in CA patients with squamous carcinomas, but not in CA patients with adenocarcinomas (Fig. 6B). In view of these differences, we conclude that LINE-1 was negatively associated with patient survival, primarily in AA patients with adenocarcinoma.

## Discussion

In this study, we established an association between patients’ racial background and immune cell profiles in lung cancer, with marked differences in immune cell infiltration and survival found between AA and CA patients with lung cancer. CA patients, but not AA patients, showed increased immune cell accumulation into the tumor microenvironment. These immune cells surrounded cancer cells only in CA patients suggesting that a “hot” versus “cold” tumor microenvironment contributes to clinical outcome differences between CA and AA patients. Our findings are consistent with several reviews examining racial differences. Nazha and colleagues (39) addressed the underrepresentation of minority ethnic groups in clinical trials and showed that AAs represent less than 4% of all patients enrolled. Kalyanaraman and colleagues (40) proposed that altered mitochondrial oxidative metabolism and differences in the tumor immune microenvironment are critical determinants of poor clinical outcomes among AA patients. Finally, Kakarla and colleagues (41) summarized the impact of racial disparities for different cancer types (prostate, breast, ovarian and uterine, digestive, urinary, and respiratory) and concluded that aggressive forms of disease and overall mortality are higher in AAs compared with other races.

Smoking is known as the leading contributor to lung cancer survival (1, 24, 25). We found significant differences of survival immune profile between AA and CA patients with different smoking histories (Fig. 3). Interestingly, among patients of the same race, comparisons between nonsmokers, former smokers, and current smokers found significant differences in CD45^+^ and CD4^+^ cells between former and current smoker CA patients, but not in AA patients. This suggests smoking status influences the immune response of CA patients more than AA patients, which is consistent with our findings that CA patients harbor a “hot tumor” environment while AA patients present a “cold tumor” environment. Moreover, within the nonsmoker subgroup, CA patients showed higher survival compared with AA patients (Fig. 3A; Supplementary Fig. S2), indicating there are other factors contributing to disease aggressiveness aside from smoking. For example, the immune profiles are distinct between CA and AA patients within the nonsmoker subgroup (Fig. 3C). Future studies will further examine other contributors to health disparities between CA and AA patients with lung cancer.

Despite the fact that AA patients have lower 5-year survival rates compared with CA patients (16% vs. 19%), female AAs had a lower incident rate compared with female CAs (49.2% vs. 57.4%), as well as a slightly lower death rate (33.3% vs. 37.9%; ref. 2). Conversely, male AAs had a higher incident rate compared with male CAs (85.4% vs. 74.3%), as well as a higher death rate (63.9% vs. 54.1%; ref. 2). In our dataset, female AA patients had comparable death rates to female CA patients (37.5% vs. 30.9%), while male AA patients had a higher death rate compared with male CA patients (55.8% vs. 41.0%). These findings are consistent with a previous report (2) and indicate that subgroup differences must be considered as critical elements in the analyses of racial and sex disparities. In this study, we revealed the association between patients’ racial backgrounds and the immune cell profiles within lung cancer. However, causal relationships are difficult to establish in human studies. Future investigations of immune cell profiles in patients with the same racial background at varying stages of disease may reveal how immune responses contribute to overall patient survival. Moreover, patients’ immunity is likely one of many factors affecting tumor progression. Other biological differences between AA and CA patients may be identified in the RNA-seq data, which can be studied more extensively in preclinical models.

Our study had two limitations that must be considered. First, larger sample sizes, especially for AA patients, must be examined in future studies. This is exemplified by the high variability of RNA-seq expression profiles in CAs compared with AAs and the lack of statistical significance noted when complex IMC profiles were stratified for race, sex, and disease stage. Second, the self-designations of race used in our study were not confirmed with ancestry admixture markers for a more accurate estimation of ancestry proportions in admixed U.S. populations.

In summary, our findings highlight key differences in immune cell profiles associated with the patients’ racial background in response to lung cancer. Therefore, future treatment plans may need to be customized to maximize the therapeutic outcome among different racial and ethnic groups.

## Supplementary Material

Supplemental Figures 1-7, Tables 1-3 Supplemental Figure 1, CD45 staining of normal adjacent tissue of 5 AA and 5 CA patients. Supplemental Figure 2, survival curves for CA and AA patients (A), from patients with different smoking histories (B), and CA and AA patients with different smoking histories (C). D, immune population comparisons between patients with different smoking histories without stratification by race. Supplemental Figure 3, survival rate (A) and curves (B) for male and female patients. C, survival curves for male and female CA and AA patients. D, immune population comparisons between male and female patients without stratification by race. Supplemental Figure 4, survival rate (A) and curves (B) patients with different tumor types. C, survival curves for CA and AA patients with different tumor types. D, immune population comparisons between patients with different tumor types without stratification by race. Supplemental Figure 5, immune population comparisons between CA and AA patients after stratifying by tumor type. Supplemental Figure 6, immune population comparisons between patients with different survival status without (A) or with (B) stratification by race. Supplemental Figure 7, immune population comparisons between patients at different disease stages without (A) or with (B) stratification by race. Supplemental Table 1, antibody list. Supplemental Table 2, number of samples processed for IMC evaluation of different tumor sub-types. Supplemental Table 3, number of samples processed for IMC evaluation of different disease stages.
