# Supplementary material for: Patients with Lung Cancer of Different Racial Backgrounds Harbor Distinct Immune Cell Profiles
Source: Cancer Res Commun. 2022 Aug 29;2(8):884–93. doi: 10.1158/2767-9764.CRC-22-0057 (PMC10010305; doi:10.1158/2767-9764.CRC-22-0057)
Supplement: Supplemental Figures 1-7, Tables 1-3 — Supplemental Figure 1, CD45 staining of normal adjacent tissue of 5 AA and 5 CA patients. Supplemental Figure 2, survival curves for CA and AA patients (A), from patients with different smoking histories (B), and CA and AA patients with different smoking histories (C). D, immune population comparisons between patients with different smoking histories without stratification by race. Supplemental Figure 3, survival rate (A) and curves (B) for male and female patients. C, survival curves for male and female CA and AA patients. D, immune population comparisons between male and female patients without stratification by race. Supplemental Figure 4, survival rate (A) and curves (B) patients with different tumor types. C, survival curves for CA and AA patients with different tumor types. D, immune population comparisons between patients with different tumor types without stratification by race. Supplemental Figure 5, immune population comparisons between CA and AA patients after stratifying by tumor type. Supplemental Figure 6, immune population comparisons between patients with different survival status without (A) or with (B) stratification by race. Supplemental Figure 7, immune population comparisons between patients at different disease stages without (A) or with (B) stratification by race. Supplemental Table 1, antibody list. Supplemental Table 2, number of samples processed for IMC evaluation of different tumor sub-types. Supplemental Table 3, number of samples processed for IMC evaluation of different disease stages. [file crc-22-0057-s01.pptx]

## Slide 1
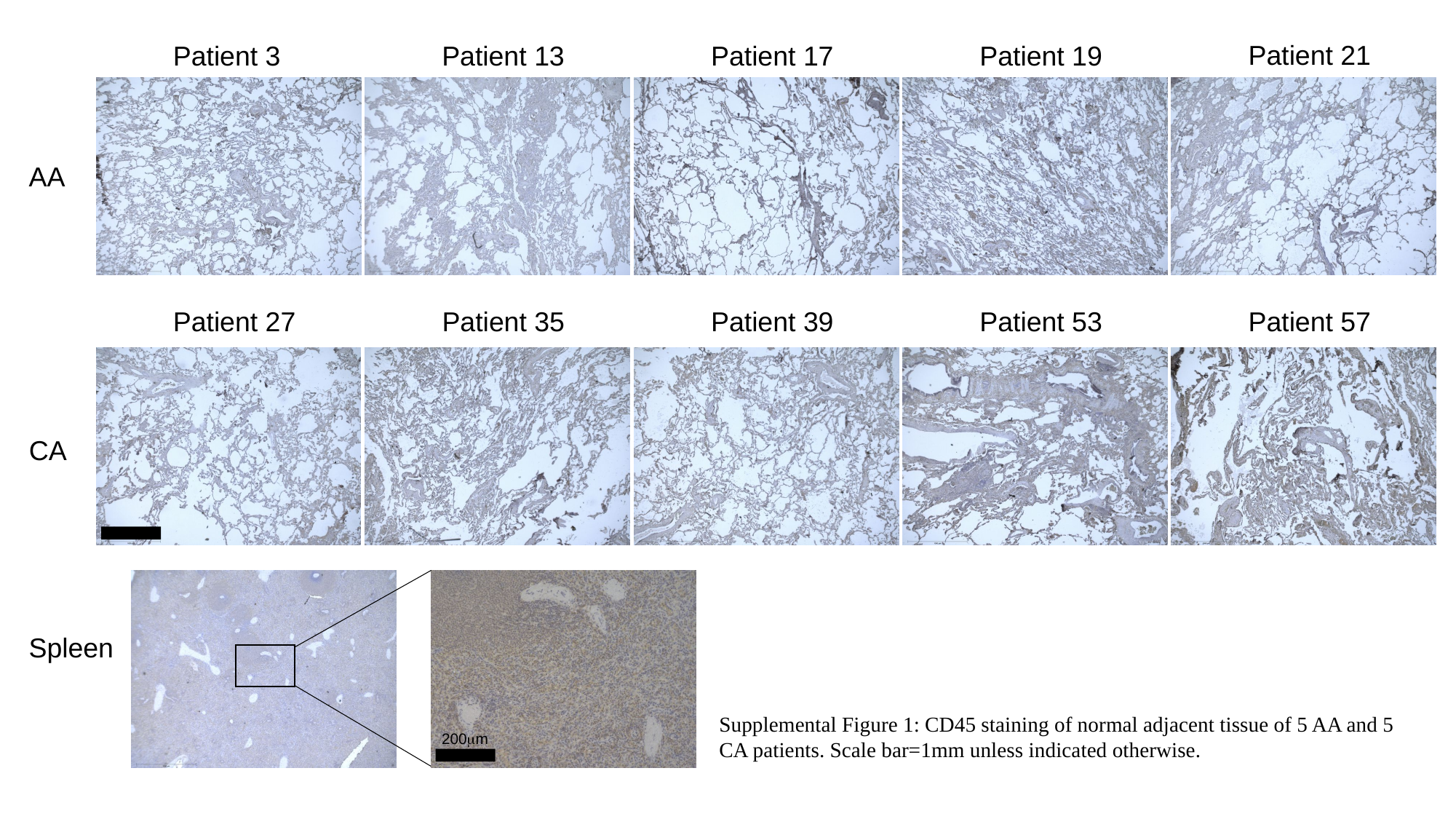

Patient 21
Patient 3
Patient 13
Patient 17
Patient 19
AA
Patient 53
Patient 57
Patient 27
Patient 35
Patient 39
CA
Spleen
Supplemental Figure 1: CD45 staining of normal adjacent tissue of 5 AA and 5 CA patients. Scale bar=1mm unless indicated otherwise.
200mm

## Slide 2
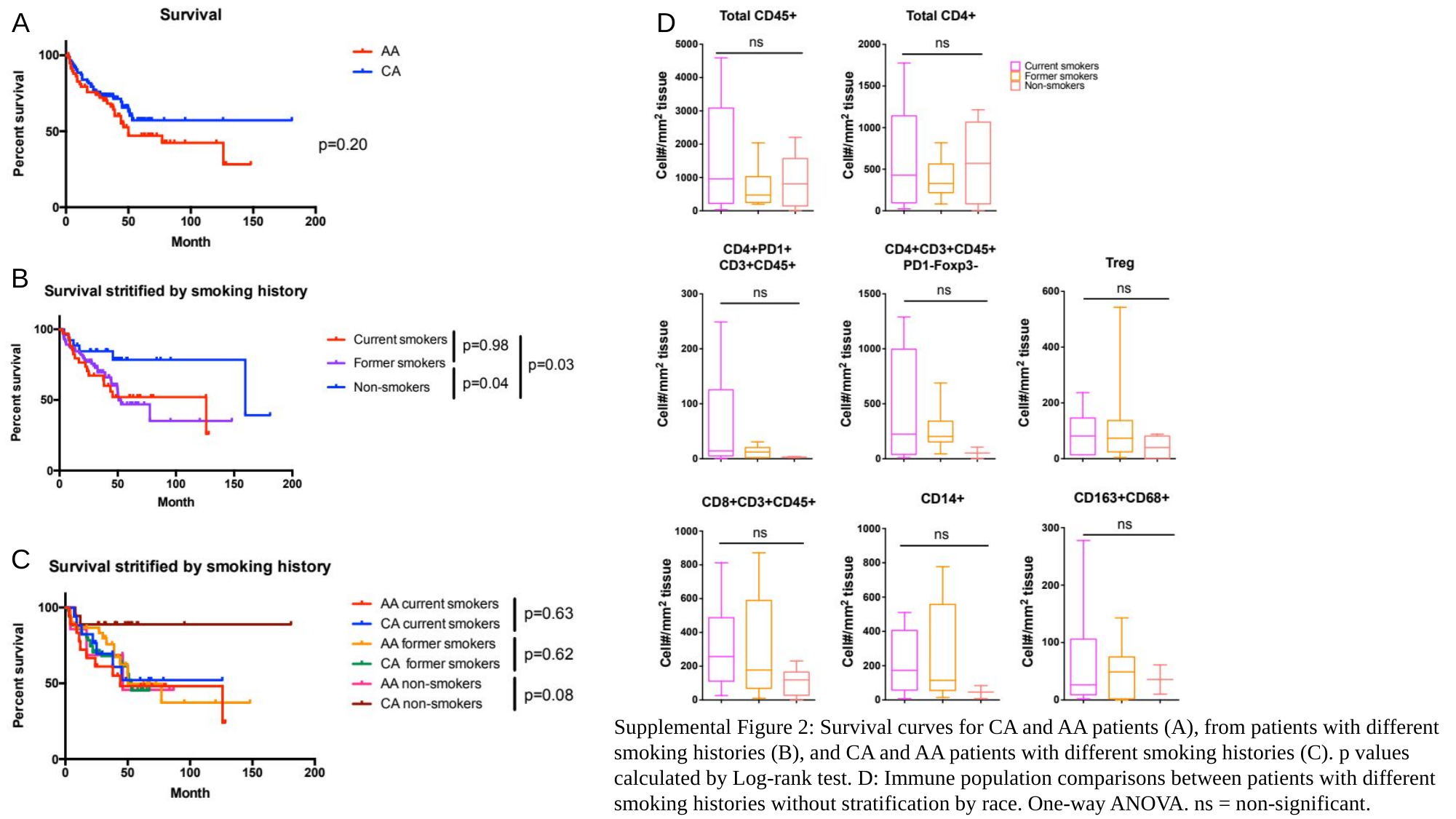

A
D
B
C
Supplemental Figure 2: Survival curves for CA and AA patients (A), from patients with different smoking histories (B), and CA and AA patients with different smoking histories (C). p values calculated by Log-rank test. D: Immune population comparisons between patients with different smoking histories without stratification by race. One-way ANOVA. ns = non-significant.

## Slide 3
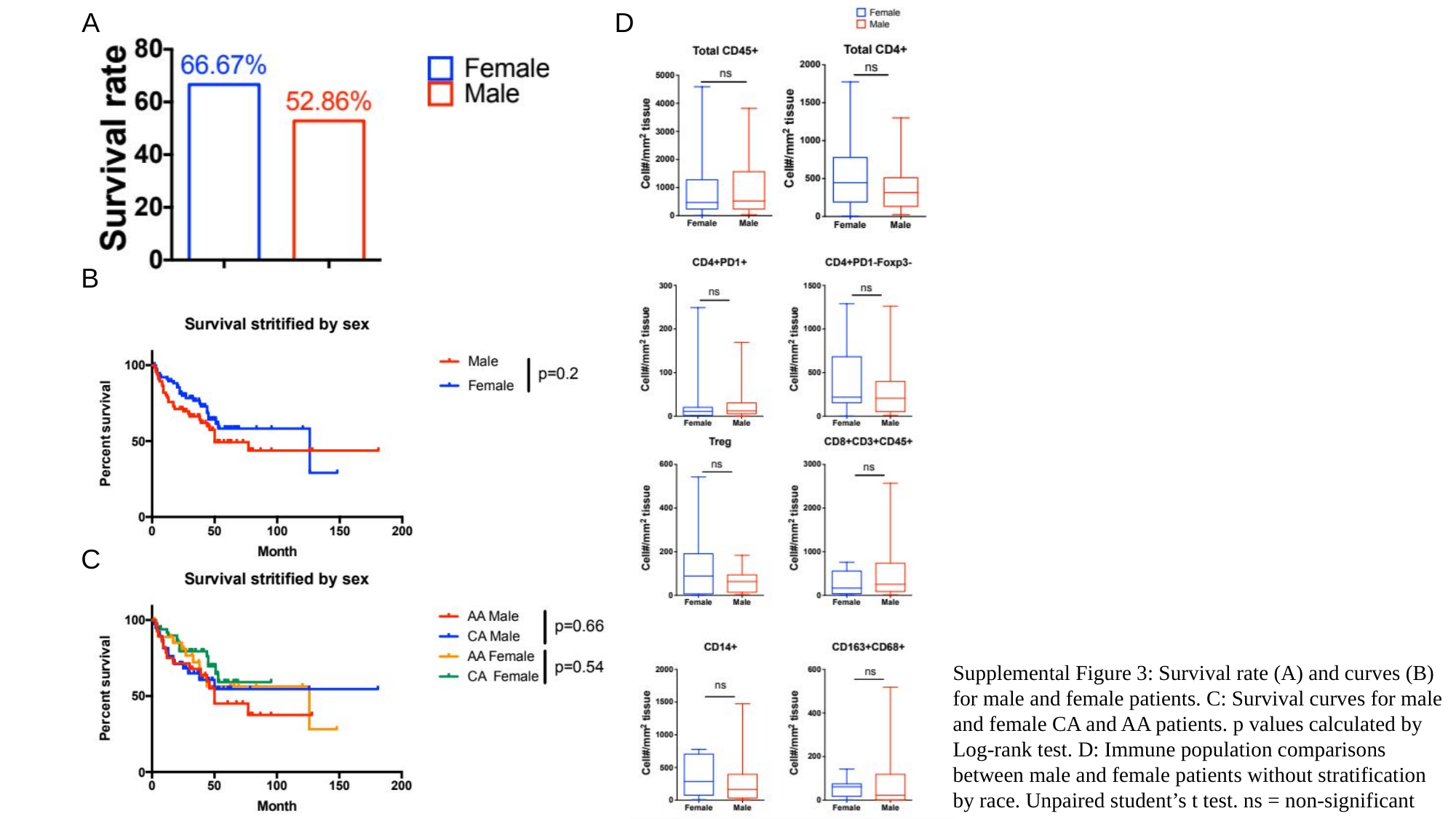

A
D
B
C
Supplemental Figure 3: Survival rate (A) and curves (B) for male and female patients. C: Survival curves for male and female CA and AA patients. p values calculated by Log-rank test. D: Immune population comparisons between male and female patients without stratification by race. Unpaired student’s t test. ns = non-significant

## Slide 4
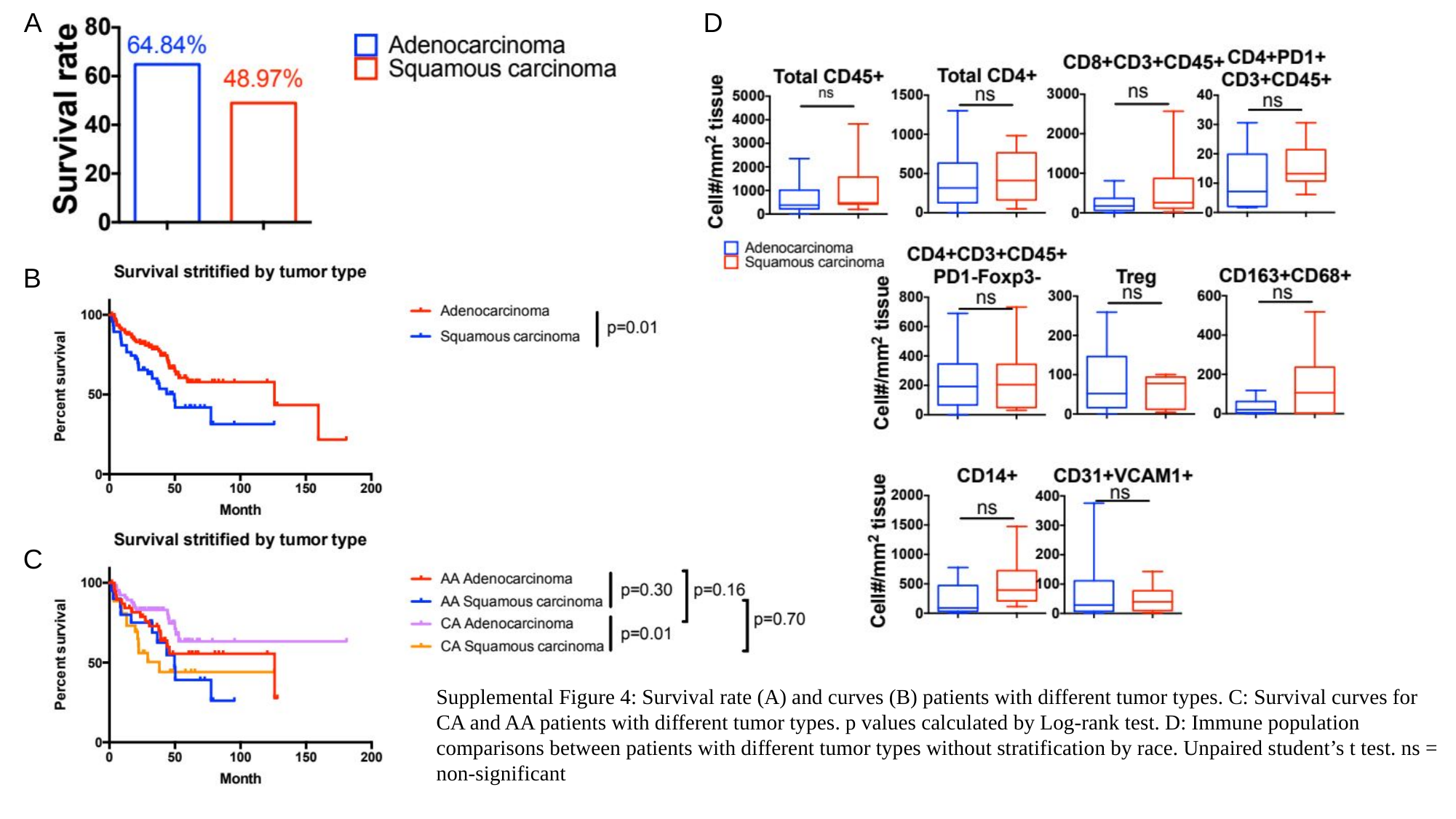

A
D
B
C
Supplemental Figure 4: Survival rate (A) and curves (B) patients with different tumor types. C: Survival curves for CA and AA patients with different tumor types. p values calculated by Log-rank test. D: Immune population comparisons between patients with different tumor types without stratification by race. Unpaired student’s t test. ns = non-significant

## Slide 5
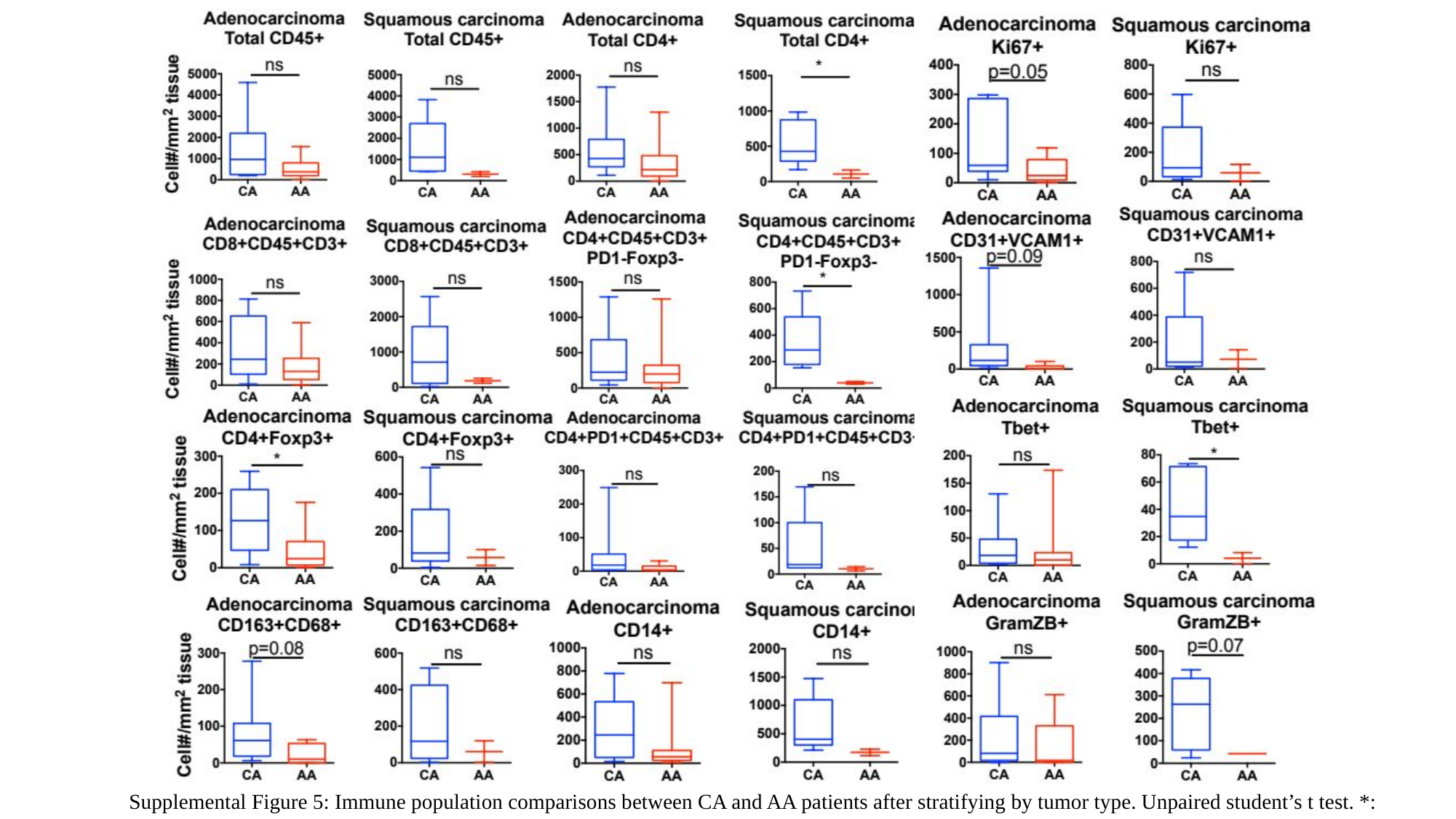

Supplemental Figure 5: Immune population comparisons between CA and AA patients after stratifying by tumor type. Unpaired student’s t test. *: p<0.05.

## Slide 6
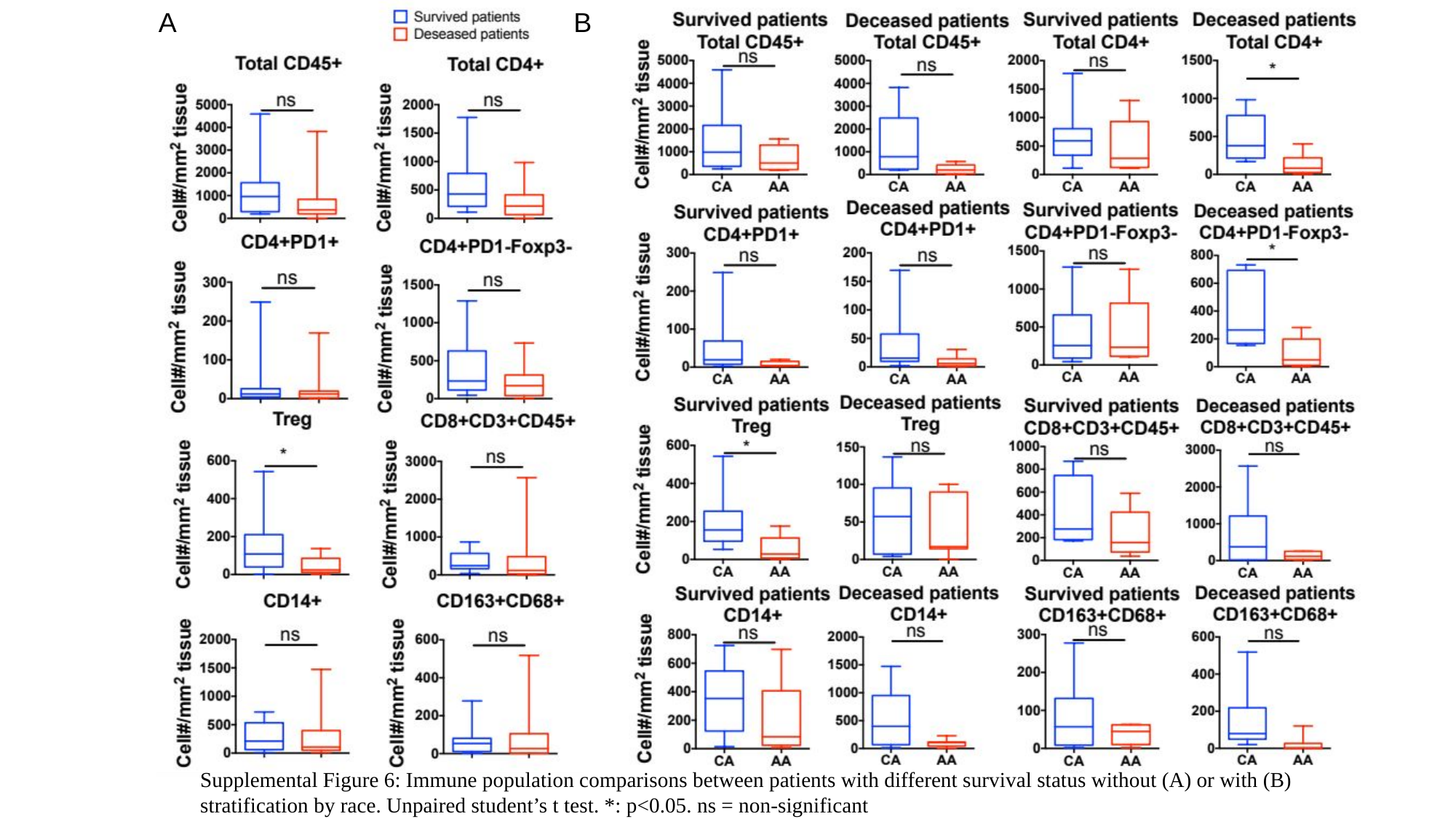

A
B
Supplemental Figure 6: Immune population comparisons between patients with different survival status without (A) or with (B) stratification by race. Unpaired student’s t test. *: p<0.05. ns = non-significant

## Slide 7
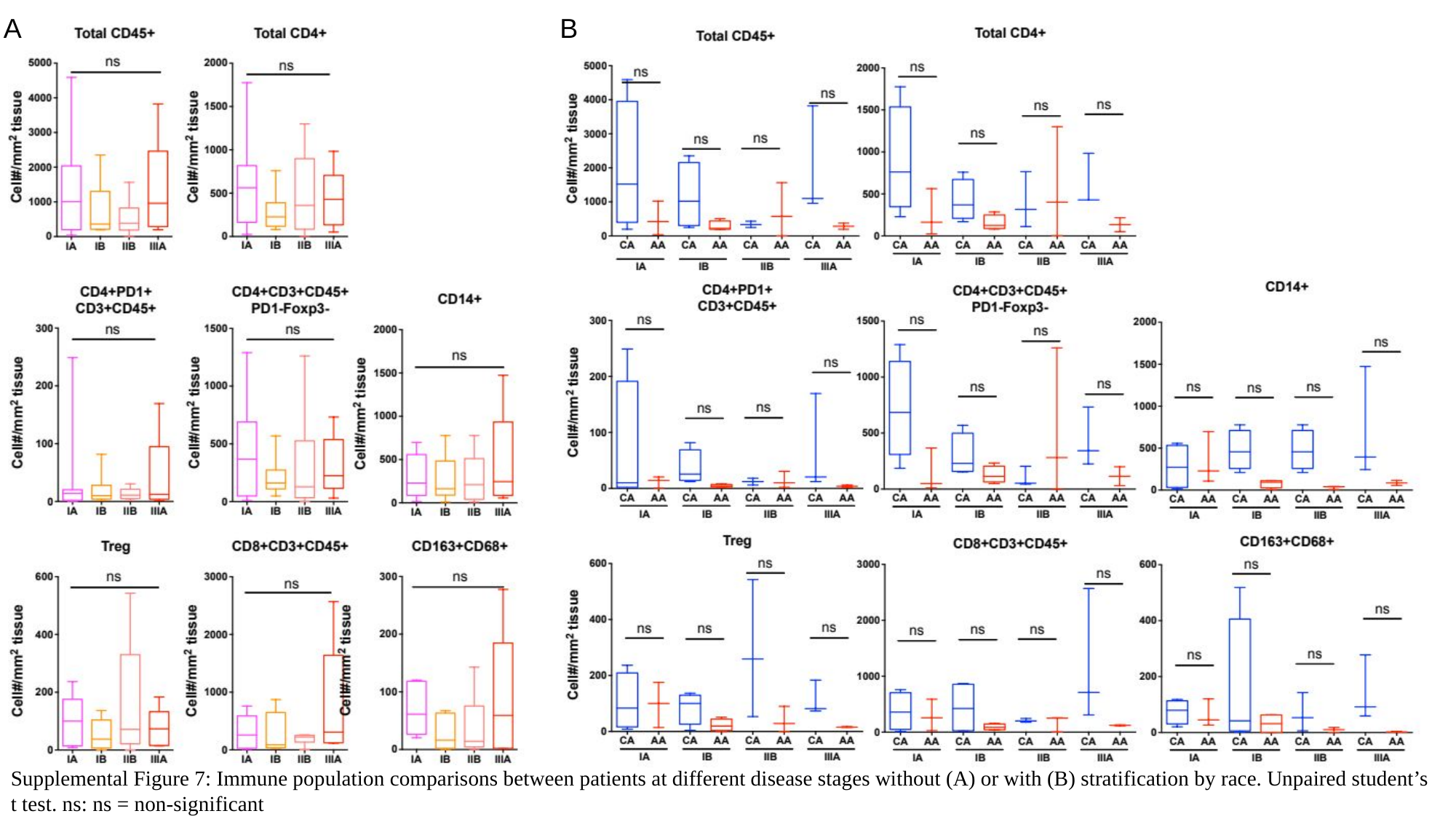

A
B
Supplemental Figure 7: Immune population comparisons between patients at different disease stages without (A) or with (B) stratification by race. Unpaired student’s t test. ns: ns = non-significant

## Slide 8
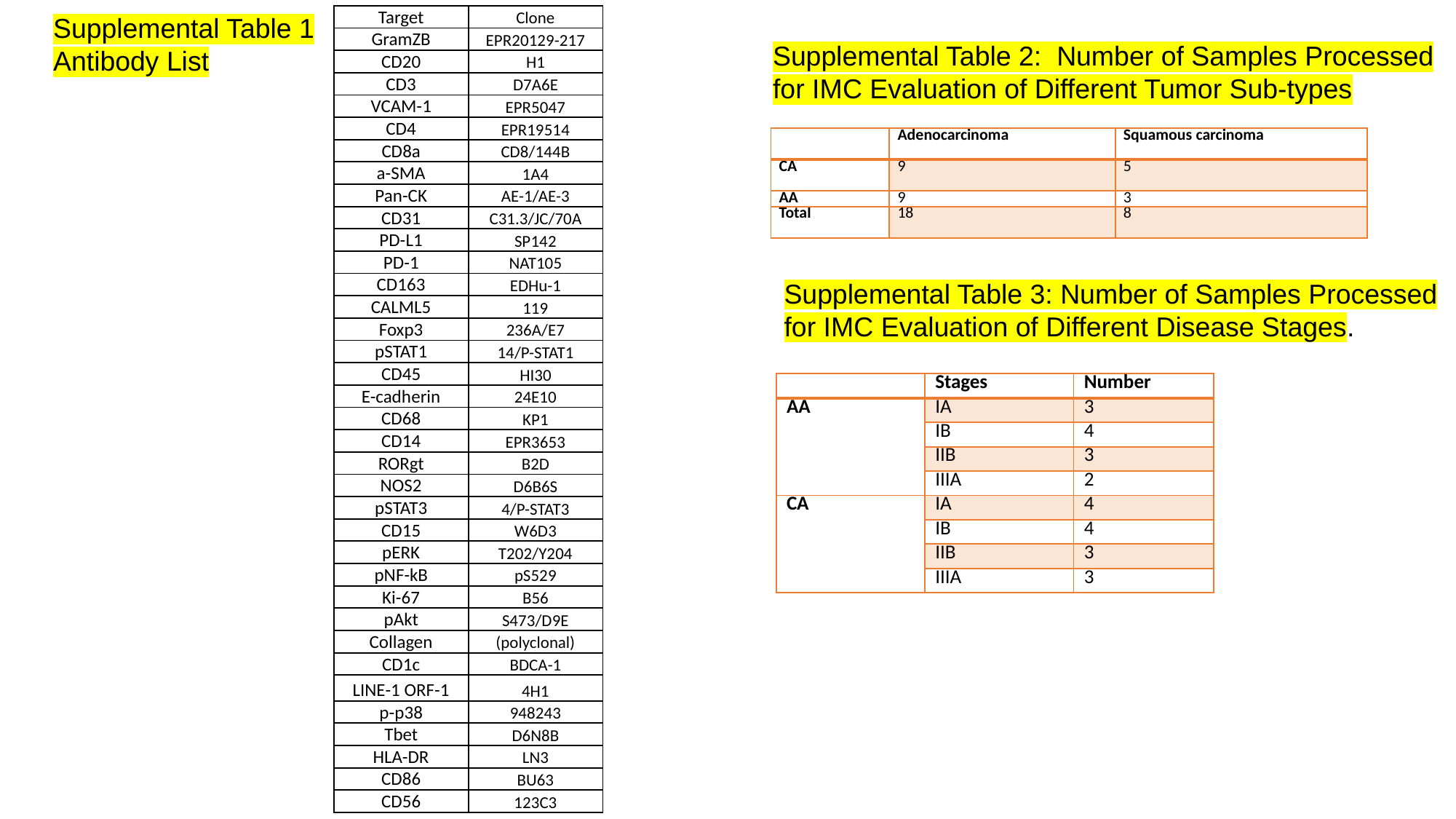

Supplemental Table 1 Antibody List
| Target | Clone |
| --- | --- |
| GramZB | EPR20129-217 |
| CD20 | H1 |
| CD3 | D7A6E |
| VCAM-1 | EPR5047 |
| CD4 | EPR19514 |
| CD8a | CD8/144B |
| a-SMA | 1A4 |
| Pan-CK | AE-1/AE-3 |
| CD31 | C31.3/JC/70A |
| PD-L1 | SP142 |
| PD-1 | NAT105 |
| CD163 | EDHu-1 |
| CALML5 | 119 |
| Foxp3 | 236A/E7 |
| pSTAT1 | 14/P-STAT1 |
| CD45 | HI30 |
| E-cadherin | 24E10 |
| CD68 | KP1 |
| CD14 | EPR3653 |
| RORgt | B2D |
| NOS2 | D6B6S |
| pSTAT3 | 4/P-STAT3 |
| CD15 | W6D3 |
| pERK | T202/Y204 |
| pNF-kB | pS529 |
| Ki-67 | B56 |
| pAkt | S473/D9E |
| Collagen | (polyclonal) |
| CD1c | BDCA-1 |
| LINE-1 ORF-1 | 4H1 |
| p-p38 | 948243 |
| Tbet | D6N8B |
| HLA-DR | LN3 |
| CD86 | BU63 |
| CD56 | 123C3 |
Supplemental Table 2: Number of Samples Processed for IMC Evaluation of Different Tumor Sub-types
| | Adenocarcinoma | Squamous carcinoma |
| --- | --- | --- |
| CA | 9 | 5 |
| AA | 9 | 3 |
| Total | 18 | 8 |
Supplemental Table 3: Number of Samples Processed for IMC Evaluation of Different Disease Stages.
| | Stages | Number |
| --- | --- | --- |
| AA | IA | 3 |
| | IB | 4 |
| | IIB | 3 |
| | IIIA | 2 |
| CA | IA | 4 |
| | IB | 4 |
| | IIB | 3 |
| | IIIA | 3 |
